# A Phase I/III, multicenter, open-label trial of talimogene laherparepvec (T-VEC) in combination with pembrolizumab for the treatment of unresected, stage IIIb-IV melanoma (MASTERKEY-265)

**DOI:** 10.1186/2051-1426-3-S2-P181

**Published:** 2015-11-04

**Authors:** Georgina V Long, Reinhard Dummer, Antoni Ribas, Igor Puzanov, Olivier Michielin, Ari VanderWalde, Robert HI Andtbacka, Jonathan Cebon, Eugenio Fernandez, Josep Malvehy, Anthony J Olszanski, Thomas F Gajewski, John M Kirkwood, Olga Kuznetsova, Lisa Chen, David R Kaufman, Jeffrey Chou, F Stephen Hodi

**Affiliations:** 1 Melanoma Institute Australia and The University of Sydney, Sydney, Australia; 2 University Hospital of Zurich, Zurich, Switzerland; 3 University of California at Los Angeles Medical Center, Los Angeles, CA, USA; 4 Vanderbilt University Medical Center, Nashville, TN, USA; 5 Centre Hospitalier Universitaire Vaudois, Lausanne, Switzerland; 6 The West Clinic, Memphis, TN, USA; 7 University of Utah Huntsman Cancer Institute, Salt Lake City, UT, USA; 8 Austin Health, Austin Hospital, Heidelberg, Australia; 9 Hôpitaux Universitaires de Genève, Geneva, Switzerland; 10 Hospital Clinic i Provincial de Barcelona, Barcelona, Spain; 11 Fox Chase Cancer Center, Philadelphia, PA, USA; 12 The University of Chicago Medicine, Chicago, IL, USA; 13 University of Pittsburgh Cancer Institute, Pittsburgh, PA, USA; 14 Merck & Co., Inc., Kenilworth, NJ, USA; 15 Amgen Inc., Thousand Oaks, CA, USA; 16 Dana-Farber Cancer Institute, Boston, MA, USA

## Background

T-VEC is a herpes simplex virus-1-based oncolytic immunotherapy designed to selectively replicate in tumors, produce GM-CSF, and stimulate antitumor immune responses. OPTiM, a Phase III trial of T-VEC vs GM-CSF in unresectable stage IIIB-IV melanoma, improved the primary endpoint of durable response rate (DRR) in the T-VEC arm (16 vs 2%).[1] Pembrolizumab, a human programmed death receptor-1 (PD-1)-blocking antibody approved for the treatment of advanced metastatic or unresectable melanoma, has demonstrated superiority over the CTLA-4-blocking antibody ipilimumab in patients with stage III or IV melanoma that received no more than one prior line of systemic therapy (PFS HR 0.58, OS HR 0.63-0.69).[2] Combining T-VEC with pembrolizumab may enhance antitumor immune responses vs either therapy alone. Here, we describe a Phase Ib/III study assessing the safety and efficacy of T-VEC + pembrolizumab in unresected stage IIIB-IV melanoma. Twenty-one patients enrolled in Phase Ib December 2014 through March 2015 at 11 institutions in Australia, Spain, Switzerland, and the United States.

## Methods

Primary objective for Phase Ib: assess dose-limiting toxicities of T-VEC + pembrolizumab. Key secondary objectives for Phase Ib: best OR, DRR, duration of response, disease control rate, PFS by investigator using modified immune-related response criteria (irRC), OS, treatment-emergent/related AEs, and potential blood/tumor biomarkers for response/resistance to combination treatment. Key eligibility criteria for Phase Ib: stage IIIB-IV melanoma naïve to systemic treatment (except adjuvant), injectable lesions, ECOG PS 0-1, no active cerebral metastases, no autoimmunity/immunosuppression, and no active herpetic infection. In Phase Ib, T-VEC is injected into cutaneous, subcutaneous, or nodal lesions at up to 4 mL of 10^6^ plaque forming units (PFU)/mL day 1, then at up to 4 mL of 10^8^ PFU/mL day 22 and Q2W. Pembrolizumab is given 200 mg IV Q2W. Treatment with both therapies continues until (whichever comes first): CR or PD per irRC, intolerance, for up to 2 yrs or, for T-VEC, when there are no longer injectable lesions. The randomized portion of the study comparing T-VEC + pembrolizumab to pembrolizumab alone was originally designed as a Phase II study. An updated Phase III design will be presented.

## Trial registration

ClinicalTrials.gov identifier NCT02263508.
